# Very bright mechanoluminescence and remarkable mechanochromism using a tetraphenylethene derivative with aggregation-induced emission†
†Electronic supplementary information (ESI) available: Details of the synthesis; structural information for the compound (NMR, IR, and mass spectra); Tables S1–S5; Fig. S1–S11. CCDC 1037840 and 1057432. For ESI and crystallographic data in CIF or other electronic format see DOI: 10.1039/c5sc00466g



**DOI:** 10.1039/c5sc00466g

**Published:** 2015-03-18

**Authors:** Bingjia Xu, Jiajun He, Yingxiao Mu, Qiangzhong Zhu, Sikai Wu, Yifan Wang, Yi Zhang, Chongjun Jin, Changcheng Lo, Zhenguo Chi, Alan Lien, Siwei Liu, Jiarui Xu

**Affiliations:** a PCFM Lab , GD HPPC Lab , Guangdong Engineering Technology Research Center for High-performance Organic and Polymer Photoelectric Functional Films , State Key Laboratory of Opto-electronic Material and Technologies , School of Chemistry and Chemical Engineering , Sun Yet-sen University , Guangzhou 510275 , China . Email: ceszy@mail.sysu.edu.cn ; Email: chizhg@mail.sysu.edu.cn ; Email: xjr@mail.sysu.edu.cn ; Fax: +86 20 84112222 ; Tel: +86 20 84112712; b State Key Laboratory of Optoelectronic Material and Technologies , School of Physics and Engineering , Sun Yat-sen University , Guangzhou 510275 , China; c Shenzhen China Star Optoelectronics Technology Co., Ltd , Guangdong , China; d TCL Corporate Research , Guangdong , China

## Abstract

Organic materials exhibiting mechanoluminescence (ML) are promising for usage in displays, lighting and sensing. However, the mechanism for ML generation remains unclear, and the light-emitting performance of organic ML materials in the solid state has been severely limited by an aggregation-caused quenching (ACQ) effect. Herein, we present two strongly photoluminescent polymorphs (*i.e.*, C_g_ and C_b_) with distinctly different ML activities based on a tetraphenylethene derivative P_4_TA. As an aggregation-induced emission (AIE) emitter, P_4_TA perfectly surmounted the ACQ, making the resultant block-like crystals in the C_g_ phase exhibit brilliant green ML under daylight at room temperature. The ML-inactive prism-like crystals C_b_ can also have their ML turned on by transitioning toward C_g_ with the aid of dichloromethane vapor. Moreover, the C_g_ polymorph shows ML and mechanochromism simultaneously and respectively without and with UV irradiation under a force stimulus, thus suggesting a feasible design direction for the development of efficient and multifunctional ML materials.

## Abstract 2

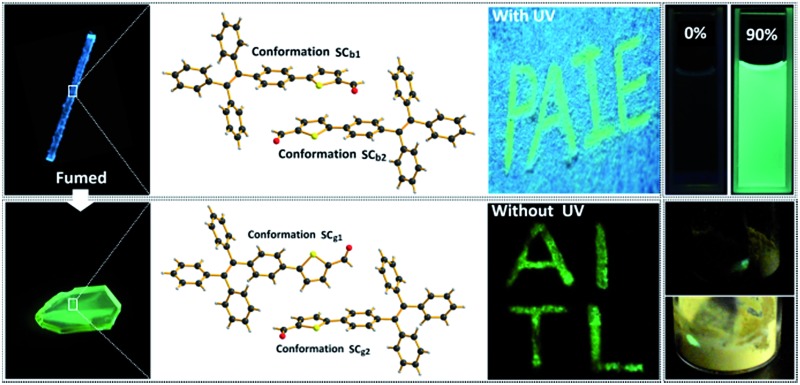
Two photoluminescent polymorphs exhibit different mechanoluminescence activities and mechanochromic behaviors.

## Introduction

The mechanoluminescence (ML) phenomenon was first found by Francis Bacon in 1605.^1^ Heretofore, research on exploiting advanced ML materials has not yet been a major focus.^2^ Materials with brilliant ML are actually of great importance from both fundamental and practical viewpoints because they are promising for usage in displays, as well as light sources and sensors.^2,3^ However, a comprehensive understanding of the crystal properties required for ML activity and the corresponding mechanisms is less well demonstrated.^4^ This lack in understanding leads to feasible design principles for these emitters, particularly those with satisfactory ML brightness, being rarely found.^5^ As reported previously, the performance of organic ML compounds can be related to both their molecular and molecular-assembly structures.^6^ Therefore, controlling the molecular arrangements in the solid state and achieving a molecular-level understanding of the relationship between the molecular conformations and packing characteristics and the resulting optical properties are the essential issues in obtaining efficient ML materials.

Notably, non-covalent intermolecular interactions, such as π–π stacking and hydrogen bonding, are important in constructing the supramolecular systems.^7^ These interactions are able to influence the final packing structure strongly, thereby making polymorphism with different ML activities more probable.^6a,8^ Nevertheless, in most cases, typical π–π stacking interactions often lead to aggregation-caused quenching, which poses significant difficulties for development of high-performance ML materials.^4b,9^ By contrast, a diametrically opposed effect was recently found to be operative in a class of chromophores with twisted conformations (*e.g.*, tetraphenylethene derivatives), which exhibit enhanced emission in the solid state with respect to the fluid solution.^10^ The discovery of this abnormal phenomenon, known as aggregation-induced emission (AIE), has sparked a rapid expansion in the field of photoluminescent sensors and electroluminescent devices.^11^ AIE also provides new possibilities for designing highly mechanoluminescent materials.

**Fig. 1:**
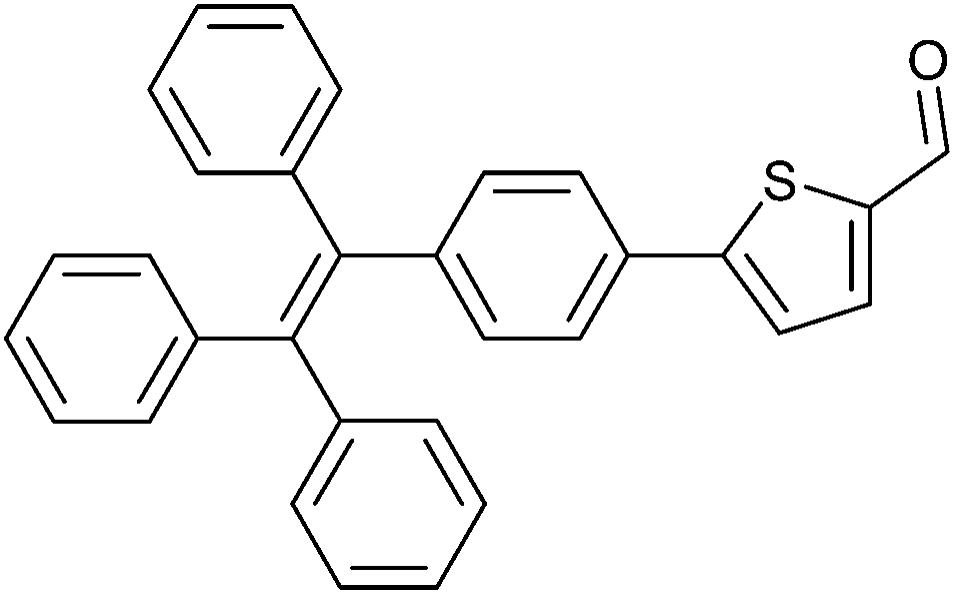
Molecular structure of P_4_TA.

This study presents a new polymorphic system that can be facilely and controllably constructed using a tetraphenylethene derivative [*i.e.*, 5-(4-(1,2,2-triphenylvinyl)phenyl)thiophene-2-carbaldehyde (P_4_TA)] as the building block (Fig. 1). The two crystalline polymorphs of P_4_TA show strong blue- and green-colored photoluminescence (PL). The blue-light crystals are significantly ML inactive, whereas the green-light ones are highly mechanoluminescent because of their distinctly different molecular packing mode and unique AIE character. The existence of polymorphs from the same molecule with exactly opposite properties provides a unique prototype to investigate the crystalline structures required for ML activity and the effect of AIE properties on ML enhancement. The relationship between the ML and the mechanofluorochromism of P_4_TA is also presented.

## Results and discussion

P_4_TA was straightforwardly prepared through a palladium-catalyzed coupling reaction by introducing 2-thiophenaldehyde to the tetraphenylethene moiety (Scheme S1†). The purified material was then characterized using nuclear magnetic resonance (NMR) spectroscopy and X-ray crystallography. The satisfactory data obtained fully confirmed its expected molecular structure (ESI†).

**Fig. 2:**
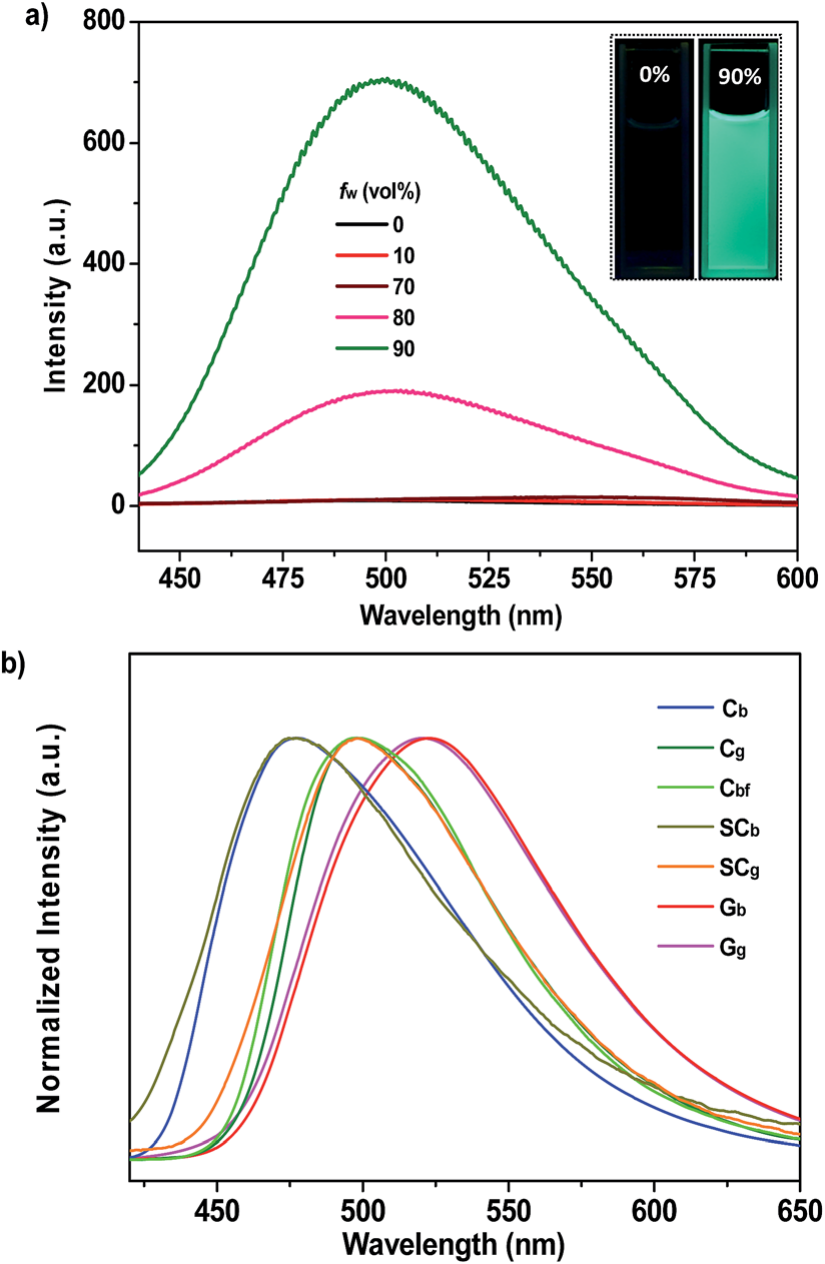
(a) PL spectra of dilute solutions of P_4_TA in water–THF mixtures with different water fractions (*f*_w_). The inset depicts the emission images of the compound in pure THF and in the 90% water fraction mixture under 365 nm UV illumination (10 μM). (b) PL spectra of P_4_TA in different phases.

The UV-visible absorption spectrum of P_4_TA was measured in a dichloromethane (DCM) solution. Two absorption bands centered at 317 and 366 nm were observed. These two bands were associated with the π–π* transition and intramolecular charge transfer, respectively (Fig. S1†). The large Stokes shift (68 027 cm^–1^) between the absorption and fluorescence spectra (*λ*_em,max_ = 513 nm) of P_4_TA in DCM was indicative of the structural difference between the ground and excited states. The resulting P_4_TA compound would probably also be AIE-active on considering that tetraphenylethene is the most frequently used AIE unit. To confirm this probability, a tetrahydrofuran (THF) solution of P_4_TA was titrated with water and the change in the fluorescence emission was monitored. P_4_TA exhibited an extremely weak emission in the THF solution, where it was well dissolved (Fig. 2a). Hence, almost no PL signal was recorded. However, when 90% (v/v) water was present, a strong green emission that peaked at 499 nm was observed and the corresponding intensity (∼705 a. u.) dramatically increased by up to ∼100 times compared to that at the 0% water fraction (∼7 a. u.). Adding water to the THF solution of P_4_TA significantly induced the formation of nanoparticles because P_4_TA molecules contain highly hydrophobic aromatic rings. In other words, the emission enhancement was caused by molecule aggregation which suggests that P_4_TA is AIE-active.^12^

**Fig. 3:**
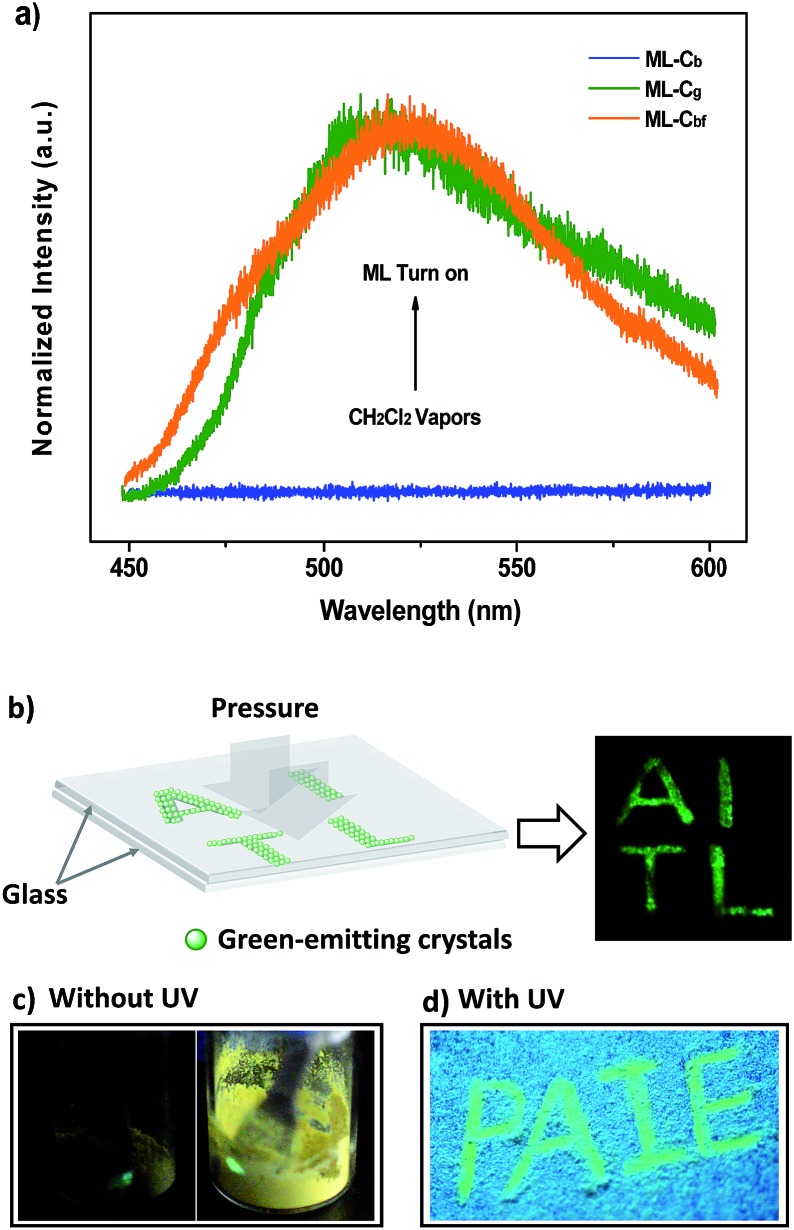
(a) ML spectra of P_4_TA in different phases. (b) An image of the capital letters ‘AITL’ being shown through ML of P_4_TA in the dark under a pressure stimulus at room temperature. (c) ML images of P_4_TA in the dark (left) and under daylight (right) at room temperature. (d) The writable mechanochromic fluorescence of P_4_TA, demonstrated using the capital letters ‘PAIE’, generated with a metal rod.

The prominent AIE character of P_4_TA motivated the application of the material in the solid state. Accordingly, block-like crystals (C_g_-form) were achieved through solvent evaporation of P_4_TA in a mixed solvent of *n*-hexane and DCM (Fig. S2a†). Compared to the emissive nanoaggregates in THF–H_2_O, the as-prepared sample C_g_ exhibited an even stronger green-light emission, centered at 498 nm [*Φ*_F,s_ = 52%] (Fig. 2b). By grinding the C_g_ crystals with a pestle or shearing them with a spatula, a very bright green light emission peaking at 517 nm was observed in the dark without UV irradiation (Fig. 3a and c and Video S1†). This experiment unambiguously illustrated that P_4_TA in the C_g_-form was ML-active. The strong ML of C_g_ was indeed clearly seen even under daylight at room temperature and was maintained while the crystals were crushed (Fig. 3c and Video S2†). Certain organic materials, such as coumarin, phenanthrene, *N*-acetyl anthranilic acid, *N*-isopropyl carbazole and *N*-phenyl imides, have also been reported to show mechanoluminescence activities. However, none of these materials could emit a ML strong enough to be observed with the naked eye under daylight at room temperature.^5,13^ The poor performance of the conventional organic ML materials should be attributed to their intrinsic ACQ property, which leads to the low emitting efficiency in the solid state. By contrast, the unique AIE feature of P_4_TA perfectly surmounted the ACQ effect and exhibited a positive effect on luminescence enhancement, thereby giving the brilliant ML of C_g_. To further demonstrate the ML characteristics of C_g_, a simple device was made by sandwiching the sample between two pieces of pre-sculptured glass. The capital letters ‘AITL’ were clearly displayed when pressure was used as the driving force, which suggests the ML ‘display capability’ of C_g_ (Fig. 3b). As such, the extraordinary AIE-active ML material P_4_TA will be a promising candidate for displays and optical recording.

**Fig. 4:**
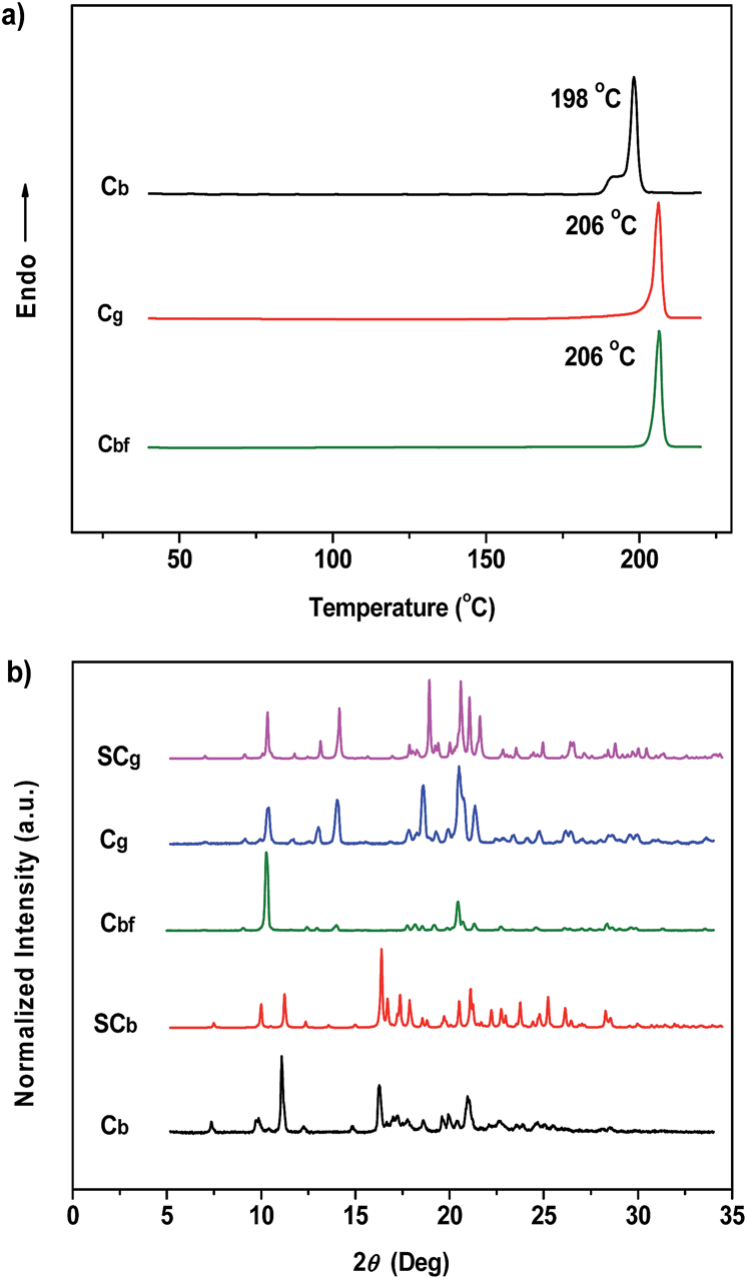
DSC curves (a) and XRD patterns (b) of P_4_TA in different phases.

Interestingly, another type of prism-like crystal (C_b_-form) was observed while exploring different processing conditions. The C_b_-form crystals, which showed an intense blue-light emission that peaked at 476 nm (*Φ*_F,s_ = 36%), could be obtained by adding ethanol into a P_4_TA/DCM solution under the action of ultrasound (Fig. S2b†). However, in contrast to the vivid C_g_ phenomenon, the P_4_TA sample completely lost its ML activity when aggregated in the C_b_-form (Fig. 3a). The C_b_ crystals showed a small melting endothermic shoulder peak at 191 °C and a sharp peak at 198 °C in the first heating curve of differential scanning calorimetry (DSC) (Fig. 4a), indicating that C_b_ was mainly composed of microcrystals that melt at 198 °C. This result is different from that of C_g_, which melts at 206 °C. Moreover, the powder X-ray diffraction (XRD) spectra also exhibited distinctly different patterns for the two samples (Fig. 4b). These results imply that the different ML activities of C_g_ and C_b_ could be attributed to their dissimilar molecular packing modes. A single crystal X-ray analysis was thus performed for the P_4_TA crystals to obtain more insight into this aspect. Single crystals of the two polymorphs (*i.e.*, SC_g_ and SC_b_) suitable for X-ray structural analysis were isolated through slow solvent evaporation of P_4_TA in mixtures of ethanol and CHCl_3_ of different concentrations.

**Fig. 5:**
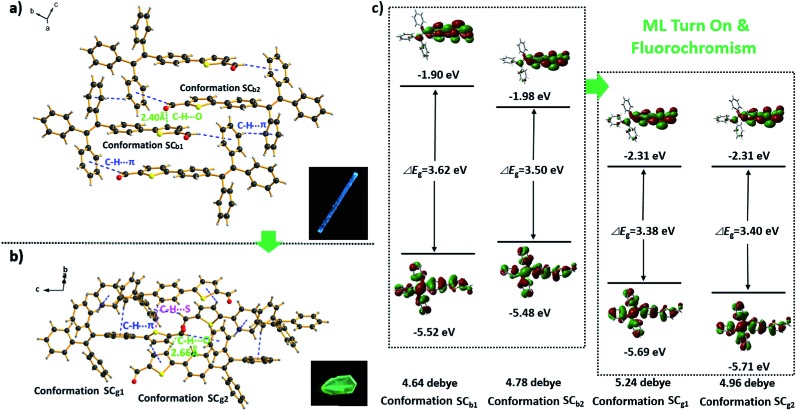
Stacking modes and intermolecular interactions of the molecules in polymorphs SC_b_ (a) and SC_g_ (b); the insets show the fluorescence images of SC_b_ and SC_g_ under an excitation of 365 nm UV light. (c) The HOMO (lower images) and LUMO (upper images) of the four conformations of P_4_TA in polymorphs SC_b_ and SC_g_ calculated at the B3LYP/6-31G(d, p) level.

The SC_g_ and SC_b_ samples emitted intense green or blue light peaked at 499 and 476 nm, respectively (Fig. 2b and 5a and b). These light emissions are similar to those of the as-prepared crystals of C_g_ and C_b_. The main peaks of the simulated XRD patterns of SC_g_ and SC_b_ also agree well with those in the patterns obtained from P_4_TA in the C_g_ and C_b_ phases, which suggests that the initial powders were mainly composed of P_4_TA microcrystals in the polymorphs of SC_g_ and SC_b_ (Fig. 4b). Further systematic analysis revealed that both SC_g_ and SC_b_ belong to the non-centrosymmetric polar space group of *P*(2)1 (Table S1†). Some previous reports have shown that dipolar structures and non-centrosymmetric molecular arrangements are favorable for obtaining piezoelectric properties, which were closely pertinent to the ML activities of the crystals.^14^ In principle, the fracture of crystals with a strong piezoelectric effect will lead to electronic discharge at the crack surface, which would result in dye excitation and generation of ML for the crystals.^5,15^ The molecular structure of P_4_TA and the crystalline symmetry of SC_g_ and SC_b_ also meet the requirements for piezoelectric properties, thus making the as-prepared crystals of C_g_ and C_b_ more active and more likely to achieve the ML character. However, the dipole moments and the HOMO–LUMO band gaps (Δ*E*_g_) of the molecules in the SC_g_ and SC_b_ polymorphs were different. These differences were caused by their distinct molecular conformations and packing characteristics. The asymmetric units in both polymorphs (*i.e.*, SC_g_ and SC_b_) were composed of two crystallographically independent molecules (*i.e.*, SC_g1_ and SC_g2_ for SC_g_, and SC_b1_ and SC_b2_ for SC_b_). Each molecule showed the formation of a C–H···O intermolecular hydrogen bond (Fig. 5a and b, S3 and S4†). Compared with SC_b_, the most notable conformational difference of P_4_TA in the SC_g_ polymorph was the dihedral angle *θ* between the thiophene and the adjacent phenyl ring. While the conformations of P_4_TA were twisted (*θ* = 18.9° for SC_g1_ and 6.1° for SC_g2_) in polymorph SC_g_, the two aromatic rings were nearly coplanar in polymorph SC_b_ (*θ* = 2.5° for SC_b1_ and 4.7° for SC_b2_) (Table S2†). In the case of SC_g_, the two thiophene rings of SC_g1_ and SC_g2_ were almost perpendicular to each other, showing a dihedral angel of 85.8°. By contrast, an anti-parallel packing mode was observed between the thiophene rings of SC_b1_ and SC_b2_ (*θ* = 3.7°) in SC_b_.

The most popular B3LYP density functional theory was then used to calculate the dipole moments and the Δ*E*_g_ of P_4_TA in the four conformations at the 6-31G(d, p) level based on their ground state geometries in the single crystals. Fig. 5c presents the results. The dipole moments of SC_g1_ and SC_g2_ in the SC_g_ polymorph were 5.24 and 4.96 debye (D), respectively. Both values were larger than those of SC_b1_ (4.64 D) and SC_b2_ (4.78 D) in SC_b_. The larger dipole moments of the molecules combining the non-centrosymmetric molecular arrangement may result in a larger net-dipole moment of the crystalline structure, and would subsequently lead to a stronger piezoelectric effect in the SC_g_ polymorph when breaking the crystals. The theoretical calculation results also suggest that the molecules in both SC_g_ and SC_b_ have ICT characteristics: the electronic transitions (mainly from HOMO to LUMO for all the four conformations in SC_g_ and SC_b_) from the occupied orbitals delocalized over the TPE (donor) moiety to the thiophenaldehyde (acceptor) moiety made major contributions to the excited states (Fig. 5c and Table S4†).^16^ SC_g1_ and SC_g2_ showed even smaller Δ*E*_g_ (HOMO → LUMO) values of 3.38 and 3.40 eV, respectively, as compared to those of SC_b1_ (3.62 eV) and SC_b2_ (3.50 eV) in the SC_b_ polymorph. The calculations showed good agreement with the solid state UV-visible spectra of C_g_ and C_b_, which absorbed at 373 nm and 367 nm, respectively (Fig. S5†). The preceding results thus indicate that the electrons of the molecules in SC_g_ can be excited with a lower energy. Hence, the stronger piezoelectric effect and the lower electronic transition energy resulted in the excitation of P_4_TA molecules and the generation of ML in the SC_g_ phase by breaking the crystals. Meanwhile, all the molecules adopted a highly twisted propeller-like conformation in the SC_g_ polymorph, which prevented the formation of detrimental species, such as excimers or exciplexes, caused by π–π stacking interactions. Furthermore, numerous intermolecular interactions such as C–H···π and C–H···S might also exist in the crystals aside from the C–H···O hydrogen bonding (Fig. 5b and Table S3†). These multiple interactions had rigidified the molecular conformations and impeded intramolecular rotation, which largely reduced the energy loss *via* non-radiative relaxation channels, and subsequently resulted in a notable AIE effect and high *Φ*_F,s_ value for P_4_TA. The preceding factors consequently made the ML of sample C_g_, which was mainly composed of P_4_TA microcrystals in the SC_g_ polymorph, highly emissive under the stimulus of mechanical force. By contrast, the weaker piezoelectric effect in the SC_b_ polymorph seemed not to reach the higher energy requirement for the electronic excitation although SC_b_ also exhibited strong photoluminescence. Consequently, P_4_TA lost its ML activity when aggregated in the C_b_ phase. These results also suggest a feasible design direction for the development of efficient ML materials through combining the prominent piezoelectric property for molecular excitation and the abnormal AIE character for emission.

The PL of C_b_ was remarkably changed when the sample was exposed to DCM or acetone vapors for about 10 min, passing from an initial blue to green light at 499 nm (C_bf_-form). The resulting spectrum of C_bf_ was superimposable on that of C_g_ (Fig. 2b). The coincidence of the PL emissions suggested that the fumed sample of C_bf_ probably had the same molecular arrangement as that of the C_g_ polymorph. Further evidence for this standpoint was provided by their similar XRD patterns and their overlapping DSC curves with the same melting point at 206 °C (Fig. 4a and b). As mentioned in the preceding discussion, sample C_g_ was ML-active. And expectantly, the ML activity of C_b_ could be tuned by simply altering the molecular packing mode upon fumigation. To verify this hypothesis, the ML spectrum of C_b_ was collected after exposure to DCM vapor (C_bf_). As anticipated, C_bf_ also exhibited a strong green light emission without UV irradiation using pressure, which revealed that the ML of C_b_ could be facilely turned on with the aid of DCM vapor. The ML emission maximum of C_bf_ was located at 520 nm, which is close to that of C_g_ (Fig. 3a).

**Fig. 6:**
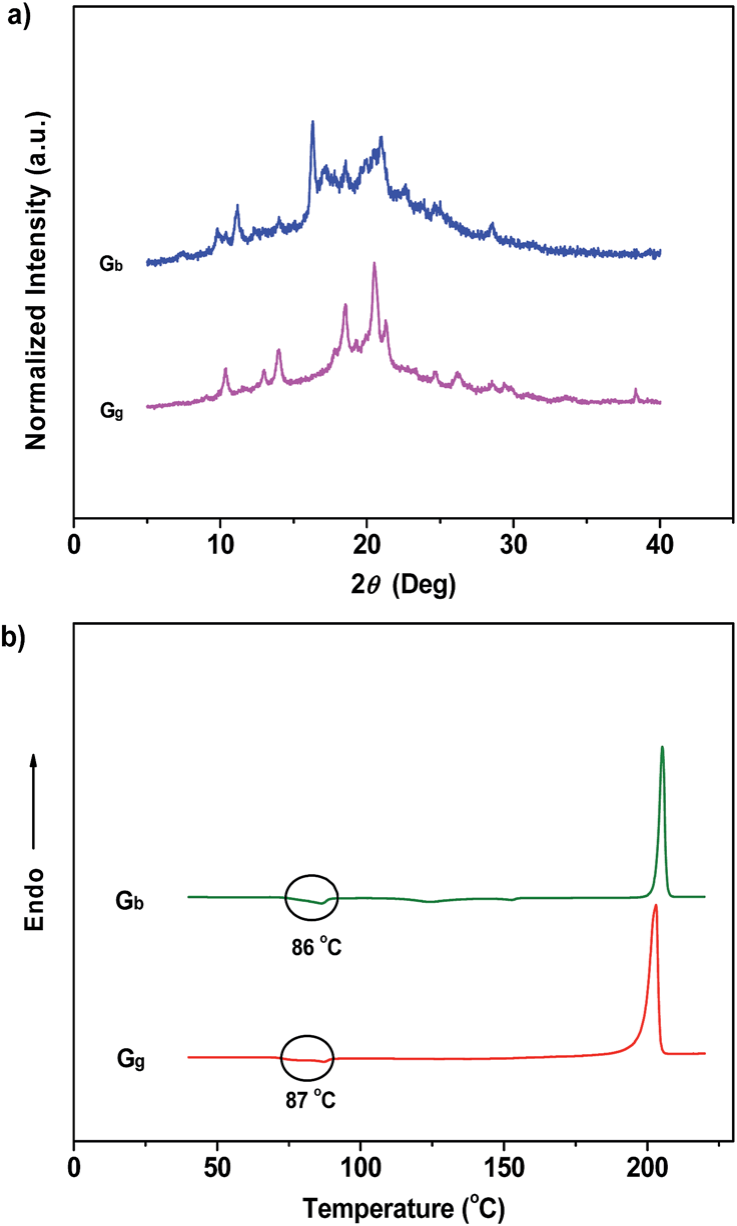
XRD patterns (a) and DSC curves (b) of the ground samples of P_4_TA: (G_b_) ground sample from the blue-light crystals; (G_g_) ground sample from the green-light crystals.

Noticeably, the ML maxima of C_g_ and C_bf_ were both significantly red-shifted (Δ*λ*_em,max_ ≈ 21 nm) as compared to their PL spectra. This result shows a special mechanofluorochromic effect. To gain an understanding of this, the influence of applied pressure on the luminescence was investigated. The PL maximum of the pristine P_4_TA in the C_g_-form shifted from 498 nm to 521 nm (G_g_) after pressing or grinding (Fig. 2a), agreeing well with its ML emission (Fig. S6†), thereby confirming that the bathochromic shift between the ML and PL of C_g_ was caused by its intrinsic mechanochromic properties. The phase characteristics of the ground sample G_g_ were determined by XRD to decipher further the relationship between the ML and the mechanochromism of C_g_. Most of the diffraction peaks were diffuse or even disappeared, although some resolvable peaks of G_g_ were consistent with those of their original crystals (Fig. 6a). This revealed that the ground sample was partially in a metastable amorphous state. Accordingly, DSC was performed for the sample after grinding (Fig. 6b). Compared with C_g_, an additional exothermal peak around 86 °C was observed in the DSC thermogram of G_g_, which demonstrated that the C_g_ crystals were partially destroyed and converted to an amorphous state by the grinding or pressing treatment. The P_4_TA molecules in the C_g_ phase adopt twisted conformations in the crystalline state to fit into the crystalline lattice, and the crystalline lattices may collapse when triggered with mechanical force. The dye molecules also then relaxed to a more planar conformation, thereby emitting redder ML and PL. In other words, the bathochromic shift of the ML for C_g_ originates from the microcrystal amorphization and the extension of molecular conjugation, which were believed to be the main reasons for the C_g_ mechanochromism.^16,17^ Unlike other conventional stimuli-responsive materials, the C_g_-form of P_4_TA can show a luminescence response and luminescence color change simultaneously and respectively both without and with UV irradiation under a force stimulus. This new kind of force-responsive material with AIE properties has not yet been achieved before, and would facilitate applications of ML materials in the field of sensors.^11*c*^ In addition, fluorescence spectroscopy was also performed to evaluate the mechanochromic behavior of P_4_TA in the C_b_ phase, and the C_b_ sample exhibited a more remarkable emission wavelength change of 47 nm upon grinding. Furthermore, the corresponding PL spectrum (G_b_) with *λ*_em,max_ = 523 nm fitted well with that for the powder ground from C_g_ (Fig. 2b), which indicated that P_4_TA could switch to the same emission under a force stimulus regardless of its initial state. Also the fluorescence ‘writability’ of C_b_ can be verified by writing on a piece of filter paper as shown in Fig. 3d. The mechanofluorochromism of C_b_ should occur by a similar mechanism to that proposed for C_g_.

## Conclusions

Based on AIE-active P_4_TA molecules, two photoluminescent polymorphs (*i.e.*, C_g_ and C_b_) with multiple molecular conformations were achieved. They can show opposing mechanoluminescence activities by tuning the molecular assembly structures in the crystals. The block-like crystals of C_g_ exhibited a very bright green color ML upon pressing or grinding under daylight at room temperature. This unique property should be attributed to the strong piezoelectric effect of the crystals and the positive effect of the AIE property on luminescence enhancement. Moreover, with UV irradiation, the C_g_ of P_4_TA showed mechanofluorochromism under a mechanical stimulus. This new kind of force-responsive compound with an AIE property could facilitate the application of ML materials in the display and sensor fields. This work may provide a feasible design direction for the development of more efficient ML materials by combining the prominent piezoelectric property for molecular excitation and the unique AIE character for emission.
