# Supplementary material for: Very bright mechanoluminescence and remarkable mechanochromism using a tetraphenylethene derivative with aggregation-induced emission
Source: Chem Sci. 2015 Mar 18;6(5):3236–41. doi: 10.1039/c5sc00466g (PMC5812443; doi:10.1039/c5sc00466g)
Supplement: Supplementary file 1 [file SC-006-C5SC00466G-s001.pdf]

Electronic Supplementary Material (ESI) for Chemical Science.
This journal is © The Royal Society of Chemistry 2015

Electronic Supplementary Information

Highly bright mechanoluminescence and remarkable mechanochromism based on a tetraphenylethene derivative with aggregation-induced emission

Bingjia Xu,[‡1,2] Jiajun He,[‡1] Yingxiao Mu,[1] Qiangzhong Zhu,[2] Sikai Wu,[1] Yifan Wang,[3] Yi Zhang,[*1] Chongjun Jin,[2] Changcheng Lo,[3] Zhenguo Chi,[*1] Alan Lien,[4] Siwei Liu,[1] and Jiarui Xu[*1]

[a] *PCFM Lab, GD HPPC Lab, Guangdong Engineering Technology Research Center for High-performance Organic and Polymer Photoelectric Functional Films, State Key Laboratory of Optoelectronic Material and Technologies, School of Chemistry and Chemical Engineering, Sun Yet-sen University, Guangzhou 510275, China.*

E-mail: ceszy@mail.sysu.edu.cn;chizhg@mail.sysu.edu.cn; xjr@mail.sysu.edu.cn; Fax: +86 20 84112222; Tel: +86 20 84112712.

[b] *State Key Laboratory of Optoelectronic Material and Technologies, School of Physics and Engineering, Sun Yat-sen University, Guangzhou 510275, China*

[c] *Shenzhen China Star Optoelectronics Technology Co., Ltd, Guangdong, China*

[d] *TCL Corporate Research, Guangdong, China*

Table of Contents

1. General experimental procedures
Materials
Characterization
2. Synthesis
Scheme S1. The synthetic route of P_4TA.
3. Figures and Tables
Fig. S1 UV and PL spectra of P_4TA in dichloromethane solution (0.1 mM).
Fig. S2 Fluorescence images of P_4TA in solid state under the illumination of 365 nm UV light.
Table S1. Single crystal information and ML activity of P_4TA in different polymorphs.
Fig. S3 Stacking mode and intermolecular interactions of the molecules in SC_b polymorph.
Fig. S4 Stacking mode and intermolecular interactions of the molecules in SC_g polymorph.
Table S2. Dihedral angles of the selected planes of P_4TA in different conformations in single crystals.
Table S3. Summarization of the supramolecular interactions in the two single crystals.
Table S4. Computed vertical transitions and their oscillator strengths and conformations.
Fig. S5 Solid state UV-Visible spectra of C_g and C_b.
Fig. S6 PL and ML spectra of P_4TA in different phases.
4. Structure Information
Fig. S7 ^{1}H NMR spectrum of P_4TA.
Fig. S8 ^{13}C NMR spectrum of P_4TA.
Fig. S9 EI-MS of P_4TA.
Fig. S10 HRMS of P_4TA.
Fig. S11 FT-IR spectrum of P_4TA.
Table S5. Crystal data and structure refinement for the two single crystals.

1. General experimental procedures

Materials 4-Bromobenzophenone, diphenylmethane, *n*-butyllithium (2.2 M, in hexane), tetrakis(triphenylphosphine) palladium(0), Aliquat 336, and 5-formylthiophene-2-boronic acid purchased from Alfa Aesar were used as received. The compound of (2-(4-bromophenyl)ethene-1,1,2-triyl)tribenzene (TPE-Br)[1] was synthesized according to the literature method. Ultra-pure water was used in the experiments. All other reagents and solvents were purchased with analytical grade from Guangzhou Dongzheng Company (China) and used without further purification. The water/tetrahydrofuran mixtures with different water fractions were prepared by slowly adding distilled water into the THF solution of the samples under ultrasonic at room temperature.

Characterization Proton and carbon NMR (^{1}HNMR and ^{13}CNMR) spectra were measured on a Mercury-Plus 300 spectrometer and a Varian INOVA500NB spectrometer, respectively ($CDCl_3$, tetramethylsilane as the internal standard). The mass spectra were measured using Thermo spectrometers (DSQ & MAT95XP-HRMS). The FT-IR spectra were obtained on a Nicolet NEXUS 670 spectrometer (KBr pellet). The UV-visible absorption spectra were determined on a Hitachi U-3900 spectrophotometer. The PL spectra were measured on an Ocean Optics Maya Pro2000with a 365 nm Ocean Optics LLS-LED as the excitation source. Light was introduced into the detector through an optical fiber. The ML spectra were collected from a spectrometer of Acton SP2750 with a liquid-nitrogen-cooled CCD (SPEC-10, Princeton) as a power detector. The thermal behaviors were determined by DSC at heating and cooling rates of 10 °C/min under N_2 atmosphere using a NETZSCH thermal analyzer (DSC 204F1). Wide-angle XRD measurements were performed at 293 K using a Bruker X-ray diffractometer (D8 ADVANCE, Germany) with an X-ray source of Cu Kα (λ= 0.15406 nm) at 40 kV and 40 mA at a scan rate of 4° (2θ)/min. The fluorescence quantum yields of solid powders were measured on an integrating sphere (C-701, Labsphere, Inc.) with a 365 nm Ocean Optics LLS-LED as the excitation source. Light was introduced into the integrating sphere using an optical fiber. The quantum chemistry calculation was performed at the B3LYP/6-31G (d, p) level of theory using the DFT method in the Gaussian 09 software.

The single crystals of P_4TA in SC_b polymorph were grown from a dilute solution, which was prepared with 5 mg sample and 4 mL $CHCl_3$/ethanol (1:3 by volume) as solvent, whereas those of P_4TA in SC_g polymorph were obtained from a concentrated solution prepared with 15 mg sample and 4 mL $CHCl_3$/ethanol (1:3 by volume) as solvent. The single-crystal X-ray diffraction data for SC_b were collected from an Agilent Technologies Gemini A Ultra system with Cu-Kα radiation (λ=1.54178 Å) at 280(10) K. The corresponding diffraction data for SC_g were obtained from a Bruker Smart 1000 CCD with Cu-Kα radiation (λ =1.54178 Å) at 150(10) K. Both structures were solved using direct methods following the difference Fourier syntheses. All non-hydrogen atoms were anisotropically refined through least-squares on F^2 using the SHELXTL program suite. The anisotropic thermal parameters were assigned to all non-hydrogen atoms. The hydrogen atoms attached to carbon were placed in idealized positions and refined using a riding model to the atom from which they were attached. The pictures of the two structures were produced using Diamond 3.2. CCDC 1037840 and 1037841 contain the supplementary crystallographic data of this paper.

1 Z. Zhao, S. Chen, J. W. Y. Lam, P. Lu, Y. Zhong, K. S. Wong, H. S. Kwok, and B. Z. Tang, *Chem. Commun.*, 2010, 46, 2221.

2. Synthesis

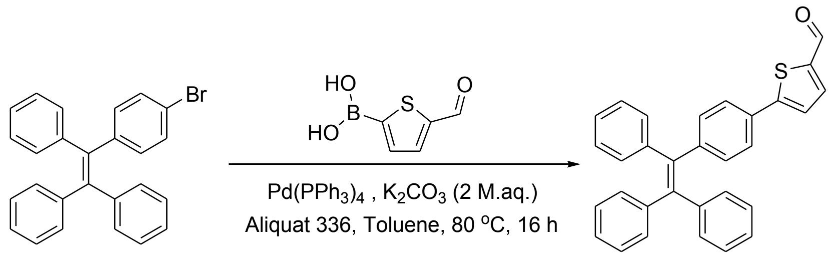

P_4TA

Scheme S1. The synthetic route of P_4TA.

5-(4-(1,2,2-triphenylvinyl)phenyl)thiophene-2-carbaldehyde (P_4TA) TPE-Br (1.20 g, 3.27 mmol) and 5-bromothiophene-2-carbaldehyde (0.75 g, 3.93 mmol) were dissolved in 30 mL toluene. Subsequently, 2 M aqueous K_2CO_3 solution (5.0 mL) and five drops of Aliquat 336 were added. The mixture was stirred for 40 min under an argon atmosphere at room temperature. The $Pd(PPh_3)_4$ catalyst was then added, and the reaction mixture was stirred at 80 ˚C for 16 h. After cooling to room temperature, the product was concentrated and purified by silica gel column chromatography with dichloromethane/*n*-hexane (*v*/*v*=1:2). The P_4TA compound was obtained as a yellow crystalline solid in 41% yield (0.59 g). ^1H NMR (300 MHz, $CDCl_3$, 25 ˚C, TMS) δ (ppm): 9.87-9.83 (s, 1 H); 7.73-7.66 (d, 1 H); 7.47-7.37 (d, 2 H); 7.36-7.30 (d, 1 H); 7.20-6.98 (m, 17 H). ^{13}C NMR (101 MHz, $CDCl_3$, 25 ˚C, TMS) δ (ppm): 182.66, 154.20, 145.31, 143.42, 143.30, 142.16, 139.97, 137.34, 132.15, 131.36, 131.32, 131.29, 130.91, 127.92, 127.85, 127.70, 126.81, 126.72, 126.67, 125.65, 123.86. FT-IR (KBr) υ (cm^{-1}): 3045, 3022, 2846, 1662, 1600, 1450, 1231, 1068, 807, 698. HRMS, *m/z*: $[M]^+$ 442.1388; calcd. for $C_{31}H_{22}OS$ 442.1391. Anal. Calc. for $C_{31}H_{22}OS$: C 84.13%, H 5.01%, S 7.25%; found: C 82.96%, H 5.14%, S 6.69%.

3. Figures and Tables

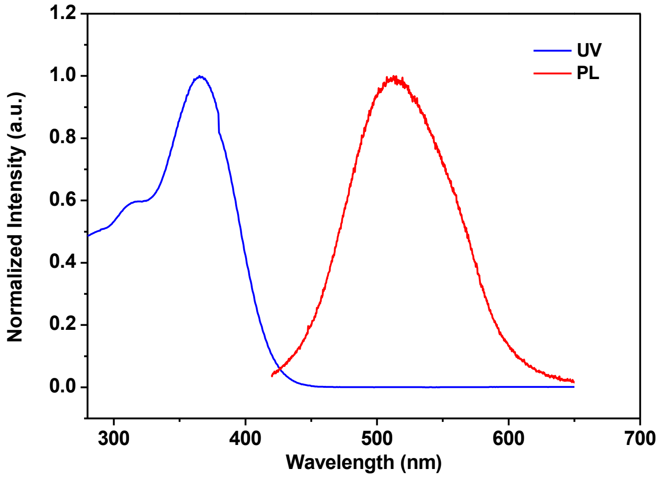

Fig. S1 UV and PL spectra of P_4TA in dichloromethane solution (0.1 mM).

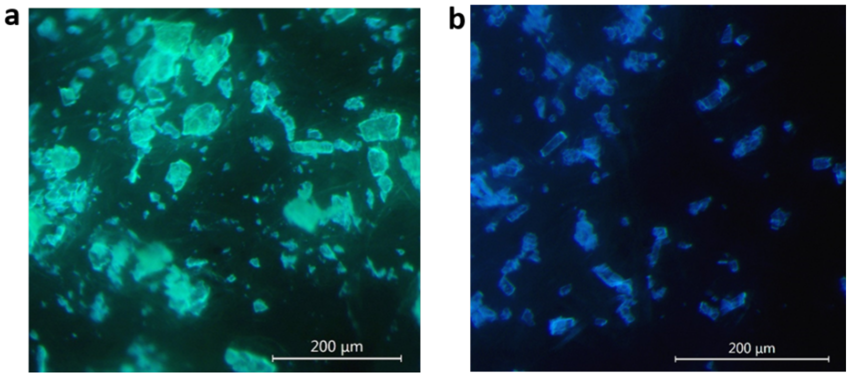

Fig. S2 Fluorescence images of P_4TA in solid state under the illumination of 365 nm UV light: (a) Crystals of P_4TA in C_g phase; (b) Crystals of P_4TA in C_b phase.

Table S1. Single crystal information and ML activities of P_4TA in different polymorphs.

Compound		Crystal system	Space Group	Symmetry	Polarity	ML activity
P_4TA	SC_b [a]	Monoclinic	*P2(1)*	Noncentrosymmetry	Polar	inactive
	SC_g [b]	Monoclinic	*P2(1)*	Noncentrosymmetry	Polar	active

[a] Blue-emitting single crystal of P_4TA. [b] green-emitting single crystal of P_4TA.

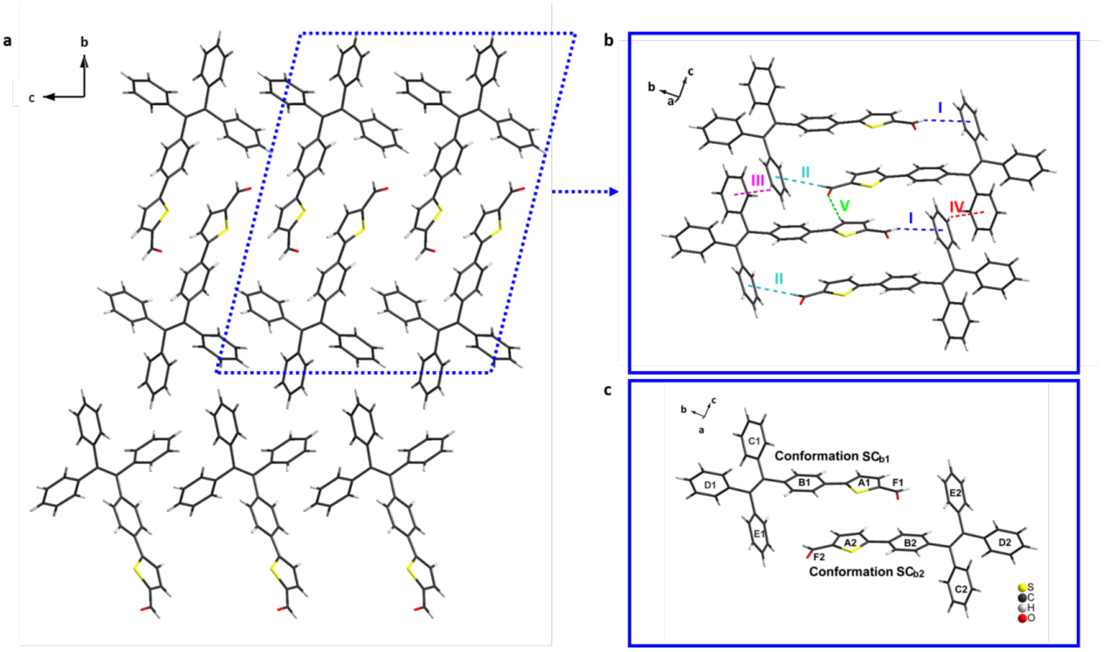

Fig. S3 Stacking mode and intermolecular interactions of the molecules in SC_b polymorph: (a) Stacking mode of the molecules of P_4TA in single crystal structure. (b) Intermolecular interactions of the molecules of P_4TA in single crystal structure. (c) The two molecular conformations of P_4TA in single crystal structure.

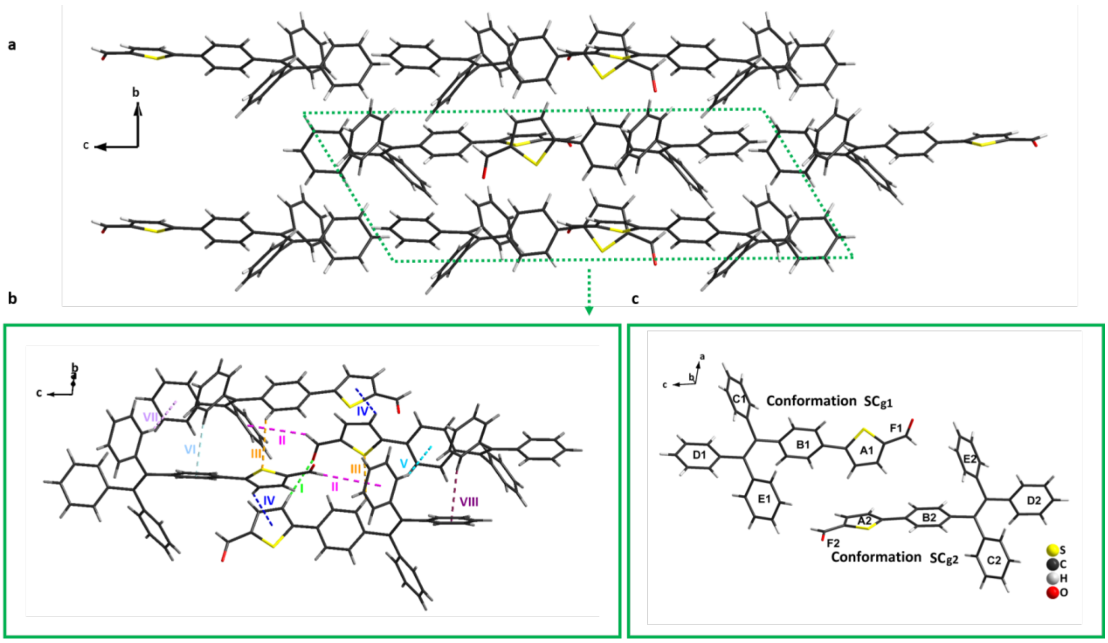

Fig. S4 Stacking mode and intermolecular interactions of the molecules in SC_g polymorph: (a) Stacking mode of the molecules of P_4TA in single crystal structure. (b) Intermolecular interactions of the molecules of P_4TA in single crystal structure. (c) The two molecular conformations of P_4TA in single crystal structure.

Table S2. Dihedral angles of the selected planes of P_4TA in different conformations in single crystals.

**Configuration**		θ / ° [a]					
		**A-F**	**A-B**	**B-C**	**C-D**	**D-E**	**A1-A2**
P_4TA	SC_{b1}	2.7	2.5	74.4	62.7	87.5	3.7
	SC_{b2}	2.0	4.7	75.1	60.3	87.3	
	SC_{g1}	6.2	18.9	82.4	53.3	75.8	85.8
	SC_{g2}	4.4	6.1	78.8	56.5	76.5	

[a] Dihedral angle. The capital letters represent the selected planes of P_4TA (see Fig. S3c and Fig. S4c). For example, 'A' represents the plane where the thiophene ring of P_4TA located. 'A1' and 'A2' represent the planes where the thiophene rings in the two molecular conformations of P_4TA located.

Table S3. Summarization of the supramolecular interactions in the two single crystals.

**Single Crystal**	**Orientation of Interaction**		**d / Å [a]**	**A / ° [b]**
**P_4TA-SC_b**	I	C-H···Ph [c]	3.2752(1)	151.120(299)
	II	C-H···Ph	3.2438(1)	140.299(359)
	III	C-H···Ph	3.0243(1)	134.209(324)
	IV	C-H···Ph	2.8980(1)	137.827(301)
	V	C-H···O	2.3994(48)	154.392(349)
**P_4TA-SC_g**	I	C-H···O	2.6600(43)	176.933(234)
	II	C-H···Ph	3.4220(1)	147.195(191)
	III	C-H···S	2.9359(8)	133.951(226)
	IV	C-H···TP [d]	2.8797(1)	132.312(186)
	V	C-H···Ph	3.0778(1)	156.733(173)
	VI	C-H···Ph	3.0767(1)	155.270(181)
	VII	C-H···Ph	3.1725(1)	155.581(173)
	VIII	C-H···Ph	3.0005(1)	154.751(181)

[a] Distance of H···O, H···S, or H···ring interaction. [b] Angel of C-H···O, C-H···S, or C-H···ring interaction. [c] Phenyl ring. [d] Thiophene ring.

Table S4. Computed vertical transitions and their oscillator strengths and conformations.

Conformation	Oscillator Strength (*f*)	Transition	Coefficients
SC_{b1}	0.5159	HOMO→LUMO	0.70205
	0.0002	HOMO-5→LUMO	0.66472
		HOMO-5→LUMO+1	0.14761
	0.4089	HOMO-1→LUMO	0.64280
		HOMO→LUMO+1	-0.27375
	0.1338	HOMO-2→LUMO	-0.18242
		HOMO-1→LUMO	0.23048
		HOMO→LUMO+1	0.60242
		HOMO→LUMO+2	0.10994
SC_{b2}	0.5159	HOMO→LUMO	0.70205
	0.0002	HOMO-5→LUMO	0.66472
		HOMO-5→LUMO+1	0.14761
	0.4089	HOMO-1→LUMO	0.64280
		HOMO→LUMO+1	-0.27375
	0.1338	HOMO-2→LUMO	-0.18242
		HOMO-1→LUMO	0.23048
		HOMO→LUMO+1	0.60242
		HOMO→LUMO+2	0.10994
SC_{g1}	0.5044	HOMO→LUMO	0.70263
	0.0002	HOMO-5→LUMO	0.65460
		HOMO-5→LUMO+1	0.19105
		HOMO-5→LUMO+3	0.11281
	0.2082	HOMO-1→LUMO	-0.20502
		HOMO→LUMO+1	0.66916
	0.2813	HOMO-1→LUMO	0.64702
		HOMO→LUMO+1	0.18935
SC_{g2}	0.4343	HOMO→LUMO	0.70267
	0.0009	HOMO-6→LUMO	-0.16918
		HOMO-5→LUMO	0.63889
		HOMO-5→LUMO+1	-0.14698
		HOMO-5→LUMO+3	0.10489
	0.3352	HOMO-1→LUMO	0.29121
		HOMO→LUMO+1	0.63678
	0.3059	HOMO-1→LUMO	0.62980
		HOMO→LUMO+1	-0.23318

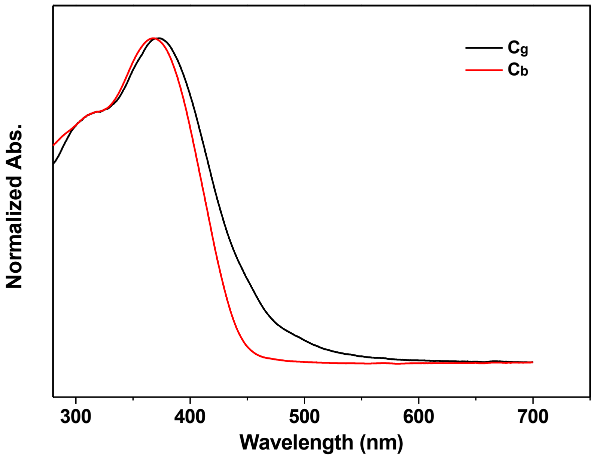

Fig. S5 Solid state UV-Visible spectra of C_g and C_b.

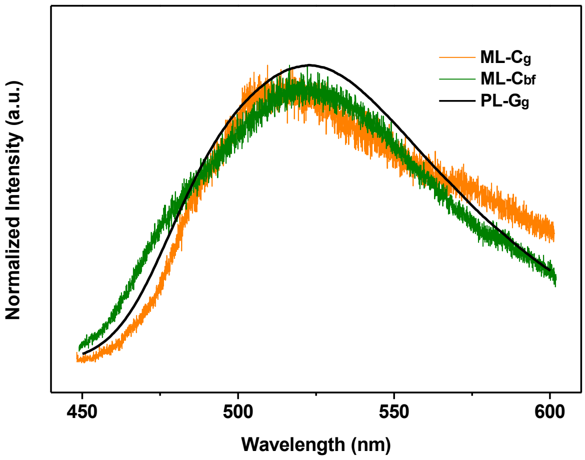

Fig. S6 PL and ML spectra of P_4TA in different phases: (ML-C_g) ML spectrum of the as-prepared green-light crystals; (ML-C_{bf}) ML spectrum of the fumed sample from blue-light crystals in dichloromethane vapors for 10 min; (PL-G_g) PL spectrum of the ground sample from green-light crystals.

4. Structure Information

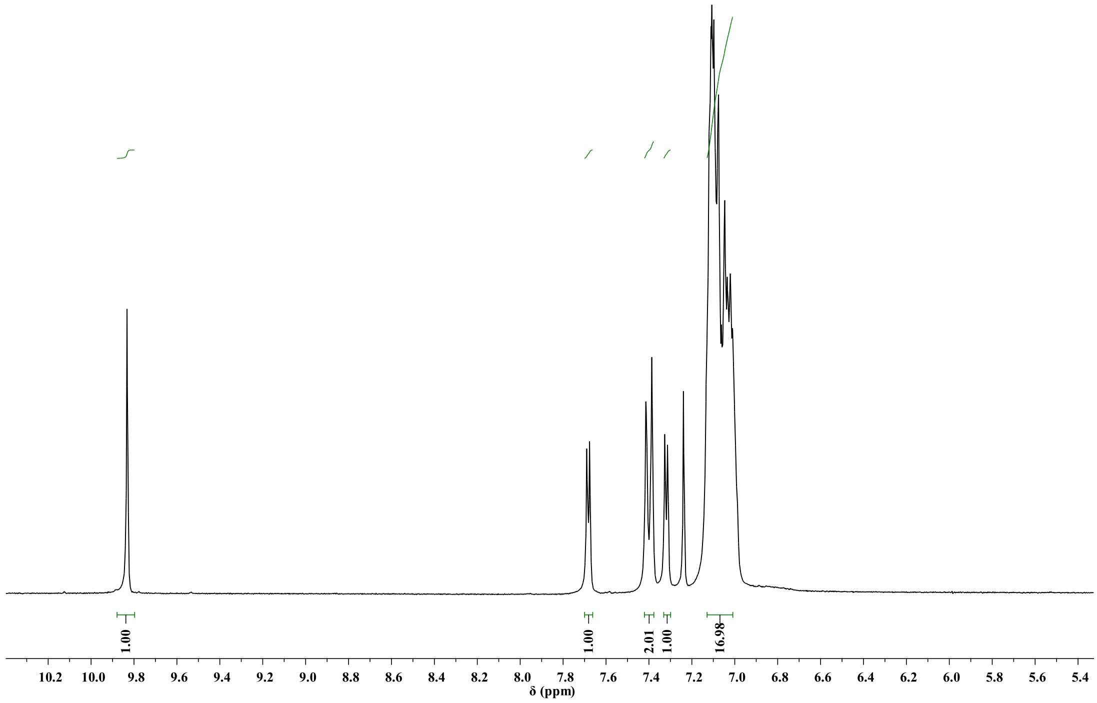

Fig. S7 ^{1}H NMR spectrum of P_4TA.

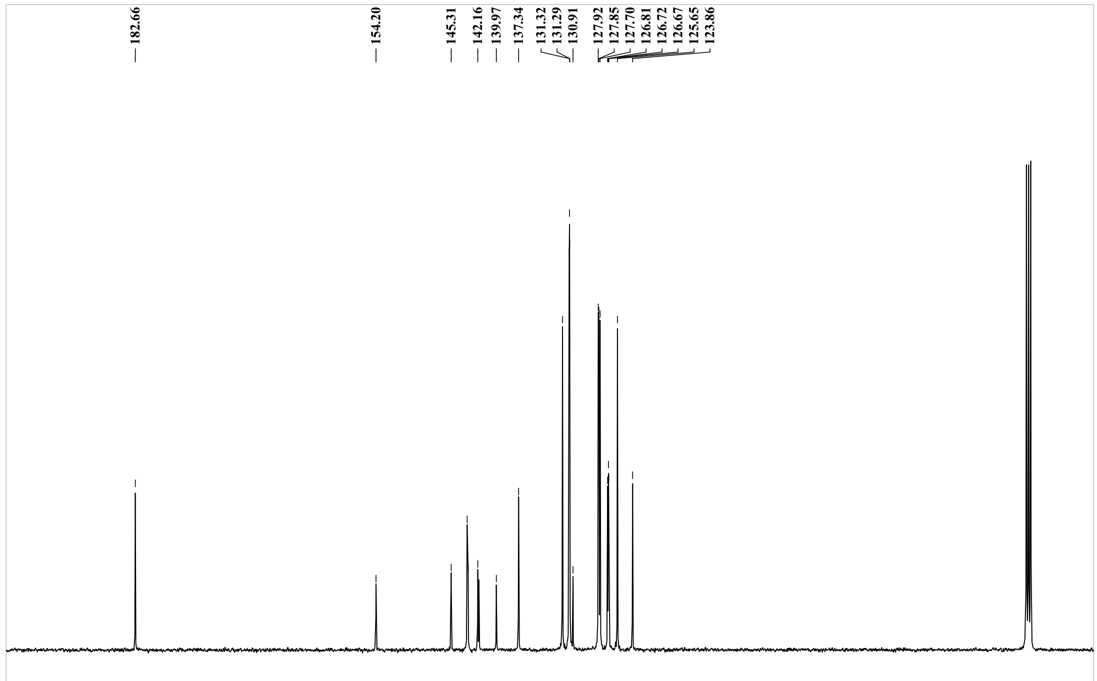

Fig. S8 ^{13}C NMR spectrum of P_4TA.

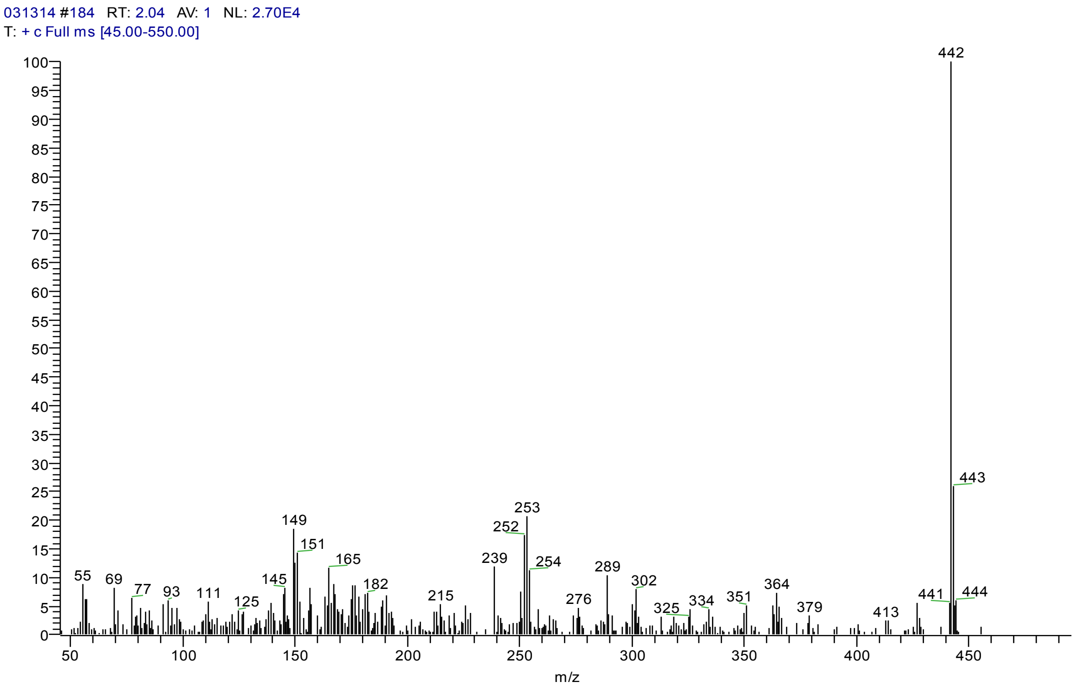

Fig. S9 EI-MS of P_4TA.

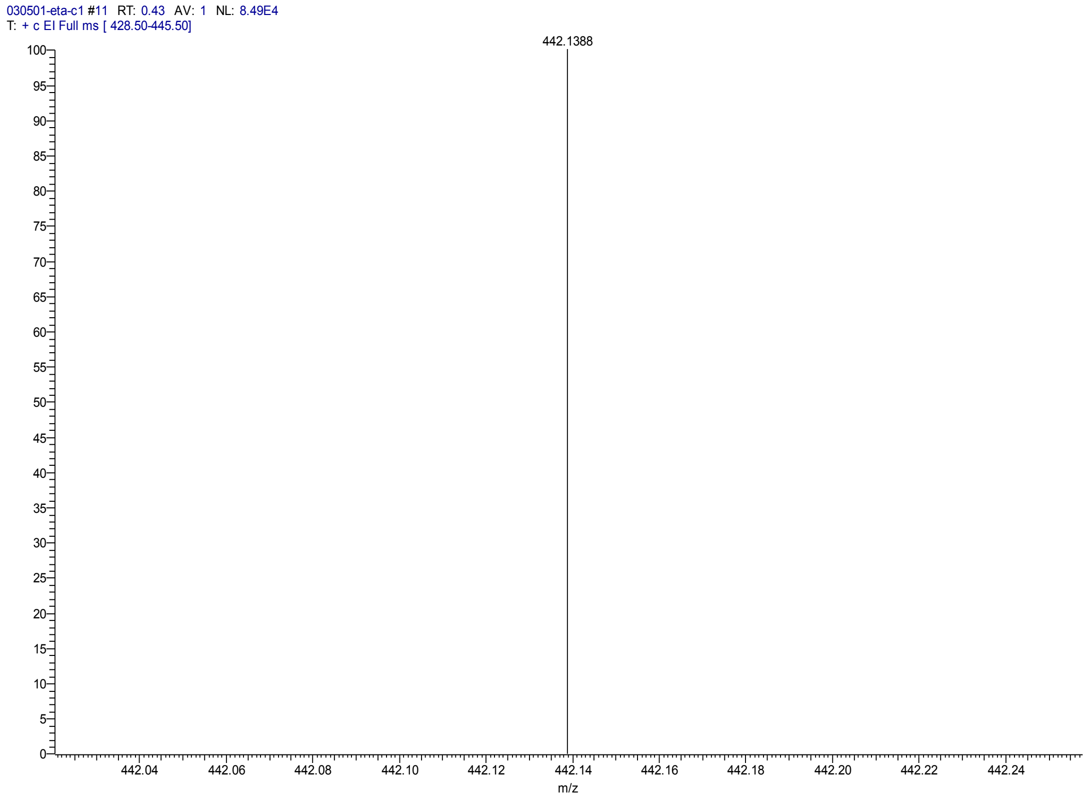

Fig. S10 HRMS of P_4TA.

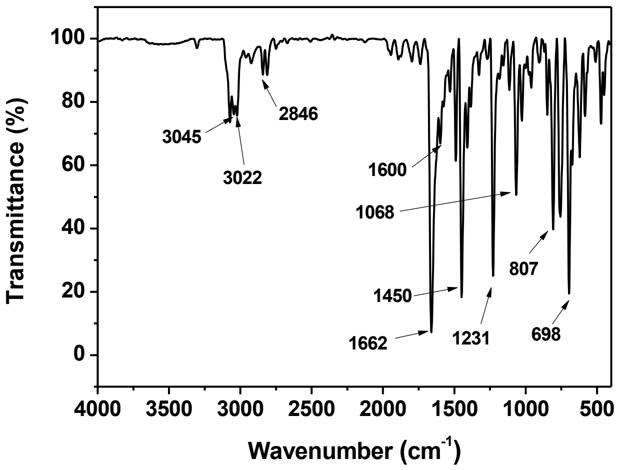

Fig. S11 FT-IR spectrum of P_4TA.

Table S5. Crystal data and structure refinement for the two single crystals.

Polymorph	SC_b	SC_g
formula	$C_{31}H_{22}OS$	$C_{31}H_{22}OS$
fw	442.55	442.55
crystal system,	monoclinic	monoclinic
T / K	280(10)	150(10)
space group	*P2(1)*	*P2(1)*
a/Å	5.5940(16)	9.8662(3)
b /Å	47.166(11)	9.3673(2)
c/Å	9.2545(3)	25.568(7)
α/ °	90	90
β/ °	103.425(3)	100.355 (3)
γ/ °	90	90
V/Å^3, Z	2375.1(12), 4	2324.55(11), 4
F (000)	928	928
crystal size / mm^3	0.30×0.10×0.10	0.10×0.10×0.10
reflections collected / unique (R_{int})	14791/7883 (R_{int} = 0.0332)	18158/7320 (R_{int} = 0.0483)
obsd reflns [$I \geq 2\sigma(I)$]	6844	6961
data/restraints/parameter	7883/1/595	7320/1/595
D_c/ Mg·m^{-3}	1.238	1.265
μ/ mm^{-1}	1.361	1.390
goodness-of-fit on F^2	1.128	1.032
R_1,[a] wR_2[b] [$I \geq 2\sigma(I)$]	0.0566, 0.1381	0.0458, 0.1188
R_1, wR_2 (all data)	0.0660, 0.1437	0.0478, 0.1206

[a] $R_1 = \Sigma||F_o| - |F_c||/\Sigma|F_o|$. [b] $wR_2 = [\Sigma[w(F_o^2 - F_c^2)^2]/\Sigma w(F_o^2)^2]^{1/2}$, where $w = 1/[^2(F_o)^2 + (aP)^2 + bP]$ and $P = (F_o^2 + 2F_c^2)/3$
